# Variation features of unfrozen water content of water-saturated coal under low freezing temperature

**DOI:** 10.1038/s41598-021-94943-6

**Published:** 2021-07-28

**Authors:** Bo Li, Laisheng Huang, Xiaoquan Lv, Yongjie Ren

**Affiliations:** 1 School of Safety Science and Engineering, Henan Polytechnic University, Jiaozuo, 454003 China; 2 Collaborative Innovation Center of Coal Work Safety and Clean High Efficiency Utilization, Henan Polytechnic University, Jiaozuo, 454003 China; 3 State Key Laboratory Cultivation Base for Gas Geology and Gas Control, Henan Polytechnic University, Jiaozuo, 454003 Henan China

**Keywords:** Hydrology, Solid Earth sciences, Hydrogeology, Mineralogy, Petrology, Tectonics

## Abstract

To determine the unfrozen water content variation characteristics of coal from the low temperature freezing based on the good linear relationship between the amplitude of the nuclear magnetic resonance (NMR) signal and movable water, pulsed NMR technology was used to test water-saturated coal samples and analyze the relationship between the unfrozen water content, the temperature and pore pressure during freeze–thaw from a microscopic perspective. Experimental results show that the swelling stress of the ice destroys the original pore structure during the freezing process, causing the melting point of the pore ice to change, so the unfrozen water content during the melting process presents a hysteresis phenomenon. When phase equilibrium has been established in the freezing process, the unfrozen water is mainly the film water on the pore surface and pore water in pores with pore radius below 10 nm. At this time, the freezing point of the water in the system decreases exponentially as the temperature increases. The micropores of the coal samples from the Jiulishan Coalmine are well-developed, and the macropores and fractures are relatively small, with most pores having a pore radius between 0.1 and 10 nm. The pore water freezing point gradually decreases with the pore radius. When the pore radius decreases to 10 nm, the freezing point of pore water starts to decrease sharply with the decreasing pore radius. When the pore radius reaches 1.54 nm, the pore water freezing point changes as fast as 600 ℃/nm.

## Introduction

Low temperature freezing of rock and soil mass is a global scientific problem. Geotechnical engineering construction in cold regions under low-temperature conditions, such as in bridge and tunnel engineering, in deposit mining, low-temperature geological storage of liquefied natural and petroleum gases, and freezing-method-based constructions, etc. These construction projects in cold regions all involve the freezing and thawing of rock and soil, and the unfrozen water content directly affects the frost heave deformation of rock and soil during the freezing and thawing process. Coal is classified as a dual porosity medium with a developed cleat system and meso/micro pores within the coal matrices^1,2^. Under the effect of low-temperature freezing, the liquid water in coal pores gradually turns to ice. However, not all the water is converted to ice mainly due to capillarity, particle surface energy, and pore pressure, leading to a change in the freezing point of water, with part of the water in the coal pores remaining liquid (called unfrozen water)^3–5^. The content and distribution of this unfrozen water directly affect the microscopic pore structure and macroscopic mechanical properties of coal after freezing. Therefore, studying the characteristics of the change in unfrozen water content of coal under low-temperature freezing condition is of great significance to low-temperature freezing engineering.

In recent years, many scholars have conducted intensive research on this topic. Currently, several methods can be used to measure the unfrozen water content, such as the ultrasonic testing method^6^, adiabatic calorimetry^7^, differential scanning calorimetry^8,9^, time domain reflectometry^10^, stereoscopy^11^, and the nuclear magnetic resonance (NMR) method^12^. The NMR technology is a widely used approach in analytical research related to coal. Li et al.^13^ used NMR to monitor the pore structure of sandstone after a freeze–thaw cycle and found that the pore size and connectivity of sandstone increases with the freeze–thaw treatment. Cheng et al.^14^ used NMR techniques to study the displacement characteristics of dense sandstone and found that dense sandstone has a multiscale pore structure dominated by pores with less than 10 μm of diameter. Using NMR techniques, Chen et al.^15^ found that more than 80% of the pores in dense rocks are sub-micropores and micro-nanopores, and movable fluid is mainly present in micro-pores with a radius greater than 1 μm. Yao et al.^16^ studied the pore structure characteristics of coal using NMR experiments and established the conversion relationship between the coal pore size and the relaxation time ($T_{2}$). Zhai et al.^17^ investigated the effect of freeze–thaw on the pore structure of coal samples and coal seam permeability using the NMR technique and found that multiple freeze–thaw cycles can greatly enhance the permeability of coal. The above research results show that NMR technology has obvious advantages in the qualitative and quantitative testing and analysis of the internal pore space of coal rocks through hydrogen-containing nuclear fluids. It also has advantages in the testing of unfrozen water content. Furthermore, the NMR test method has the advantages of low external interference in the determination of water distribution and migration, the short test time requirement, high accuracy, directness and non-destructiveness in applications over other methods for testing the content of unfrozen water^18,19^.

Most of the research on unfrozen water content focused on permafrost and rock areas. Li et al.^20^ conducted freeze–thaw cycle experiments on soil columns at controlled laboratory ambient temperatures of − 15 °C to 15 °C and found that liquid water remains at the end of the freezing period, with the unfrozen water content between 0.07 and 0.10 m^3^ m^−3^. Kozlowski^21^ proposed a semi-theoretical and -empirical formula for predicting the unfrozen water content in frozen soil. Qin et al.^22^ used the thermodynamic theory of continuous media to derive a theoretical formula for the relationship between unfrozen water content and temperature. Zhang et al.^23^ developed a new model to describe the variation of volumetric unfrozen water content in frozen soils, validated experimentally. Panday et al.^24^ found that water migration in saline areas is accompanied by solute migration, which affects the freezing temperature and the unfrozen water content. Huang et al.^25^ established a theoretical equation between the unfrozen water content and freezing temperature in low-temperature rocks using the distribution function of rock pore volume. The unfrozen water content can be expressed as an exponential form of freezing temperature. Huang et al.^26^ investigated the unfrozen water content, ice pressure and frost heave deformation of low temperature saturated rocks by building a thermo-hydro-mechanical coupling model of freezing rock under low temperature. Liu et al.^27^ investigated the variation of the unfrozen water content and the damage characteristics of rocks during freezing by using the CT image histogram technique. In summary, the research on unfrozen water content focused mainly on the mathematical model of unfrozen water content, and relatively few studies have been conducted on the relationship between the unfrozen water content, pore structure, and water freezing point of coal rocks.

In this work, NMR technology has been used to study the characteristics of the unfrozen water content during the freeze–thaw process and understand the relationship between temperature, pressure, and freezing point during the ice-water phase change of a coal mass. Considering the pore volume ratio of different pore sizes inside the coal and based on thermodynamic theory, the critical freezing threshold of the pore water inside the coal under low-temperature freezing conditions is obtained.

## Experimental work

### Mechanism of measuring unfrozen water content by NMR

A proton bears a slight magnetic moment originating from its spin and thus can be viewed as a micro magnet. In a porous material within a fixed external magnetic field, all the protons will tend to align along with the direction of the magnetic field, resulting in magnetization. The magnitude of magnetization is proportional to the number of protons and inversely proportional to the temperature according to the Curie law^28^. The NMR mechanism aims to analyze the response of hydrogen protons in the medium in the magnetic field through the vibration of a hydrogen-containing proton substance, producing a measurable amplitude called the relaxation time. For different ores of hydrogen-containing protons in the medium, the relaxation times form different relaxation time spectra, which are mainly divided into longitudinal relaxation time ($T_{1}$) and transverse relaxation time ($T_{2}$). The relaxation behavior of hydrogen-containing fluids in porous media can be analyzed by transverse relaxation time $T_{2}$^29^.

$T_{2}$ is defined as the time required for the transverse magnetization vector of the hydrogen-containing proton substance to reach 37% of the amplitude maximum from the amplitude maximum. The $T_{2}$ relaxation times is closely related to the free-induction decay (FID) curve of protons in the porous medium and thus the $T_{2}$ distribution can be obtained from the FID curve by applying the Fourier transformation. The peak value of the FID is directly proportional to the number of protons in the material. Hence the FID peak measurements can provide a convenient detection for the number of H_2_O molecules, i.e., the amount of water, contained in the porous material. Kim et al.^30^ experimentally verified the good linear relationship between the amplitude of the NMR signal and the movable water content in the measured substance.

The Transverse relaxation time $T_{2}$ follows the following exponential relationship^31^:

1 $$ M(t) = M(0)\exp \left( { - \frac{t}{{T_{2} }}} \right), $$

where $t$ is the time, $M(t)$ is the transverse magnetization vector at *t* after the onset of hydrogen proton relaxation, and $M(0)$ is the maximum transverse magnetization vector at the onset of hydrogen proton relaxation.

The expression of transverse relaxation time $T_{2}$ can be derived from Eq. (1):

2 $$ \frac{1}{{T_{2} }} = \frac{\ln [M(0)] - \ln [M(t)]}{t}. $$

Consequently, the magnitude of $T_{2}$ is influenced by two factors: (1) the effect of temperature change on $T_{2}$, and (2) the change of the hydrogen proton content of the substance. Moreover, transverse relaxation time $T_{2}$ can be used to describe the pore distribution of the coal sample, and its distribution curve is consistent with the pore size distribution curve^32^. Therefore, the distribution curve of $T_{2}$ can be used to analyze the pore water distribution and content in the coal samples, and the variation characteristics of the unfrozen water content of the coal samples during the freeze–thaw process can be obtained.

### Experiment procedure

#### Coal sample preparation

The coal samples used in the experiment were anthracite coals from the No. II-1 coal seam, Jiulishan Coal Mine, Jiaozuo, China. Six standard cylindrical coal samples with dimensions of Φ25 mm × 50 mm were drilled from the raw coal by using a liquid nitrogen coring device, and a hole of 5-mm diameter with a depth of 25 mm was designed and drilled in the middle of each coal sample. The coal samples were placed in a muffle furnace, dried at a constant temperature of 80 ℃, and weighed every 24 h. When the difference between two records was less than 0.01 g, a coal sample was considered fully dried, and the weight of the coal sample was recorded as $m_{0}$.

The dried coal samples were fully saturated with water within a negative pressure satiation device, and then were weighed every 24 h. When the difference between two records was less than 0.01 g, the saturation was considered complete. The water-saturated coal sample was placed in a constant-temperature and -humidity cabinet for moisture balance, the temperature was set to room temperature (25 ℃), and the coal sample was weighed every 4 h during the process of moisture balance. The process generally took 3–5 days. The coal sample was weighed repeatedly until its weight was nearly unchanged, which is regarded as the completion of moisture balance, and the weight of the coal sample was recorded as $m_{1}$. The calculation formula for the coal sample moisture content is as follows: $w = (m_{1} - m_{0} )/m_{0}$.

#### Coal sample freezing process

The prepared coal samples were wrapped with a fresh-keeping film and placed in a low-temperature incubator for freezing at − 20 ℃ for 12 h to reduce the effect of moisture in the air on the moisture content of the coal mass during the freezing process. After freezing, the coal samples were taken out. A SH700-8 data recorder was used to collect the center temperatures of the coal samples and check whether they are fully frozen.

#### $T_{2}$ distribution

**Figure 1:**
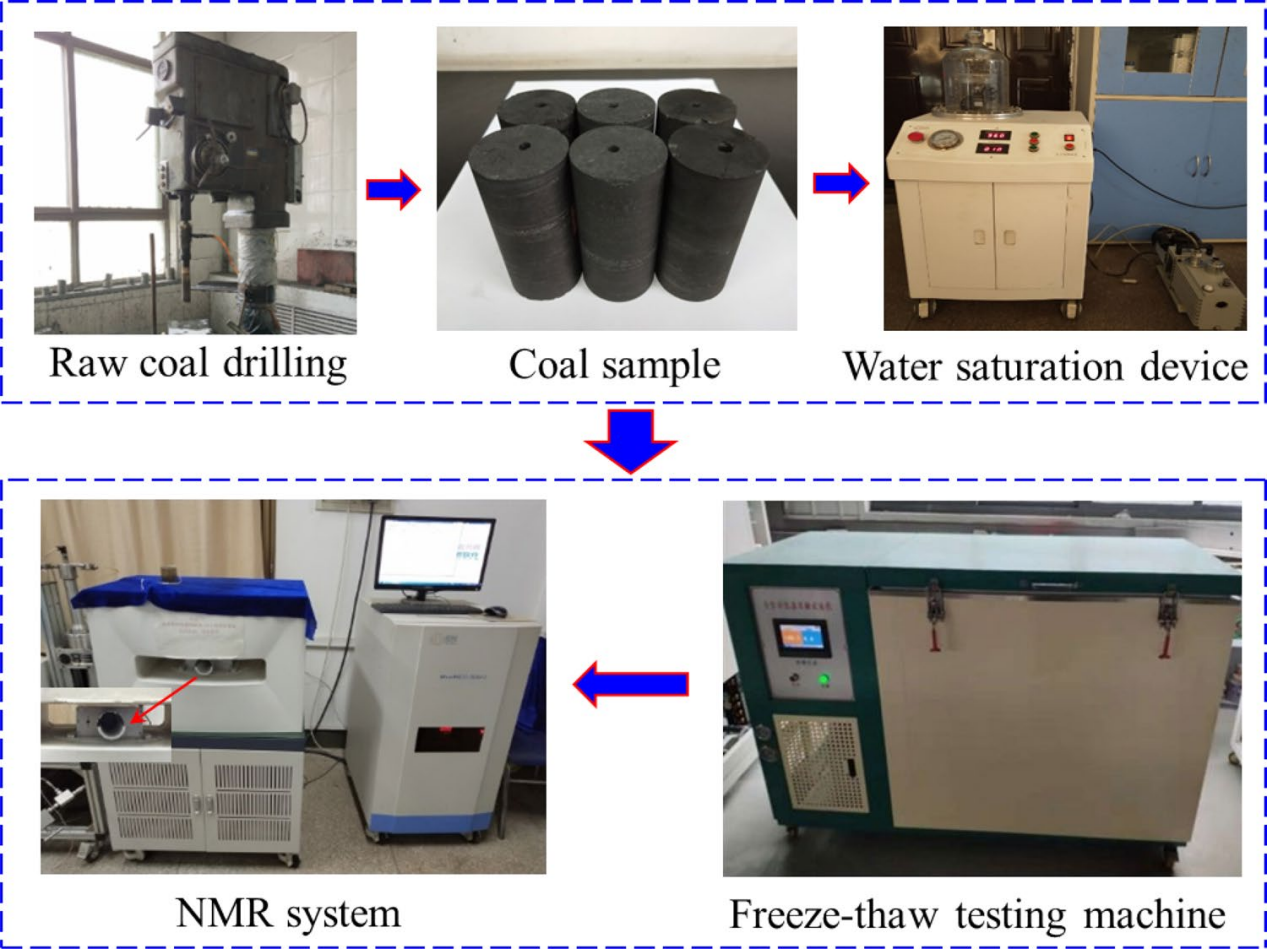
System diagram of NMR experiment.

After the temperature inside the sample tubes reached the targeted temperature and stabilized, the frozen coal samples were placed into in the sample tubes, while the NMR signals were immediately collected, and the strengths of the NMR signals and the freezing temperatures were recorded. After the test, the coal samples were placed in the low-temperature incubator whose temperature was adjusted downward, between 0.5 and 5 ℃. Two hours after the temperature measurement, the internal temperatures of the coal samples were checked to determine if they reached equilibrium. The above steps were repeated to measure the strength of the nuclear magnetic signal at different temperatures from − 20 to 20 ℃. The NMR experimental system diagram is illustrated in Fig. 1.

## Results and discussion

**Figure 2:**
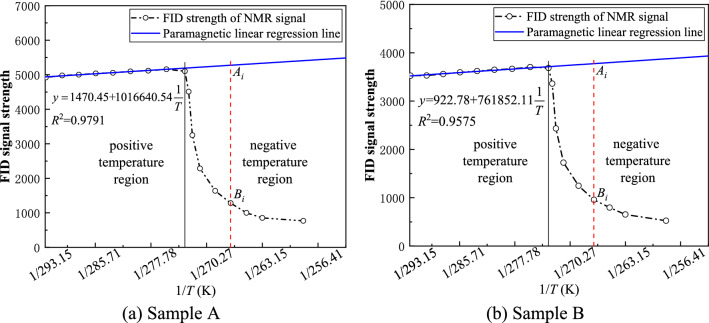
Variation of FID signal strength of coal samples with temperature.

The NMR signal amplitudes of the pore water in the six groups of coal samples under different freezing temperatures were obtained through the NMR experiments. A certain similarity was observed through comparison, and all coal samples were taken from the same large coal block under the same experimental conditions. Thus, this paper only shows two groups of coal samples (i.e., coal samples A and B) with the largest difference in test results for comparison. The experimental data of the two groups of coal samples are shown in Table 1 and are plotted in Fig. 2.

**Table 1:** FID signal amplitudes at different freezing temperatures.

Freezing temperature (K)	FID signal strength of sample A	FID signal strength of sample B	Temperature region (K)	FID signal strength of sample A	FID signal strength of sample B
253.15	707.9	509.2	273.15	5101.1	3685.1
258.15	768.9	525.5	275.65	5158.8	3703.4
263.15	854.9	654.3	278.15	5116.9	3667.2
265.15	1000.5	796.3	280.65	5097.6	3648.3
267.15	1282.0	957.9	283.15	5056.8	3620.9
269.15	1639.4	1248.8	285.65	5038.0	3594.2
271.15	2286.9	1725.9	288.15	5003.5	3559.1
272.15	3250.3	2434.6	290.65	4978.9	3529.5
272.65	4512.3	3361.7	293.15	4922.4	3525.7

The NMR technology can evaluate the unfrozen water content in a frozen sample by measuring the FID. Because the FID value is proportional to the amount of liquid water in the specimen, the unfrozen water content in the frozen sample can be calculated^33,34^. Figure 2 shows the change curve of the FID signal strength of the coal samples with temperature. In the positive temperature region, the moisture in the sample does not start to freeze, and the moisture in the coal will not change. But with the decrease of temperature, the FID signal increases gradually, which conforms to the principle of Curie’s law. According to the measured data, the corrected paramagnetic linear regression line is obtained by fitting the signal in the positive temperature zone, and the paramagnetic linear regression line is extended to the negative temperature zone intersecting the ordinate^35,36^. In the negative temperature-freezing region, the amplitude of the NMR signal decreases sharply with the decrease of temperature, indicating that the unfrozen water content in the coal sample decreased sharply.

### Calculation of unfrozen water content

**Figure 3:**
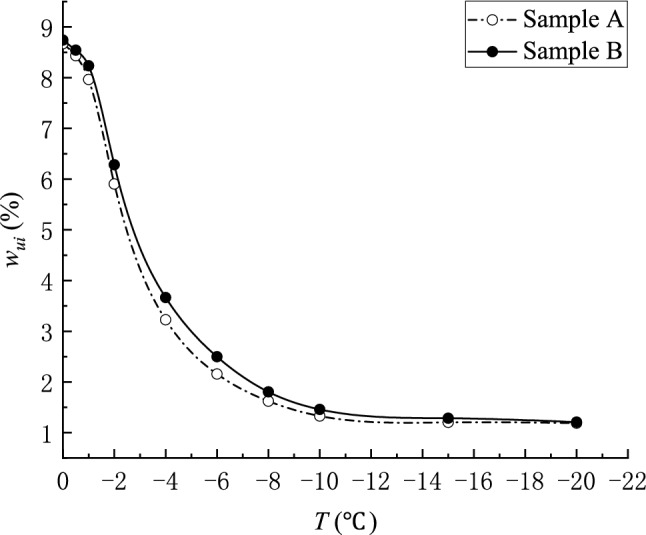
Unfrozen water content—temperature curve during freezing of coal samples.

As shown in Fig. 2, the FID decreases linearly with increasing temperature in the positive temperature region. Thus, the unfrozen water content in coal can be characterized by the amplitude change of the FID signal measured at different freezing temperatures. The formula of the unfrozen water content in coal can be expressed as Eq. (3)^37–40^.

3 $$ w_{ui} = \frac{{A_{i} }}{{B_{i} }}w_{w0} , $$

where $w_{ui}$ is the unfrozen water content at temperature $i$, %; $A_{i}$ is the measured signal strength at temperature $i$; $B_{i}$ is the signal intensity value corresponding to a linear regression line function at temperature $i$; and $w_{w0}$ is the initial saturated water content. Using the coal sample moisture content formula, the initial saturated water contents of coal samples A and B were calculated as 8.75% and 8.86%, respectively. Using this method, the unfrozen water content in the negative temperature-freezing region of the experimental coal sample is calculated, and the relationship between the unfrozen water content and the freezing temperature is shown in Fig. 3.

Figure 3 shows that the unfrozen water content in the coal decreases gradually with the temperature, but a part of the liquid water always remained unfrozen. This phenomenon occurred because the freezing point of the pore water in the process of ice water transformation in the coal is related to pressure, temperature and pore size, thus the ice water transformation process finally reached the equilibrium state.

### Relationship between temperature, pressure and freezing point during ice water phase change

In water freezing, hydrogen bonds are formed between water molecules. Given the directionality of the hydrogen bond, large gaps are formed during the clumping of water molecules, which causes the volume expansion of water after freezing. The expansion of the water freezing volume in the coal mass leads to an increase in pore pressure, which limits the formation of hydrogen bonds between water molecules. Given that the process of water forming into ice is restrained, the phase transition of ice and water reaches a state of two-phase equilibrium according to the Clausius–Clapeyron equation^41^:

4 $$ \frac{dP}{{dT}} = \frac{L}{T\Delta v}, $$

where $dP/dT$ is the rate of change of pressure with temperature; $L$ is the enthalpy of phase change, J/kg; $T$ is the temperature, K; $P$ is the pressure, Pa; and $\Delta v$ is the specific volume change of the phase change latent heat, m^3^/kg.

**Figure 4:**
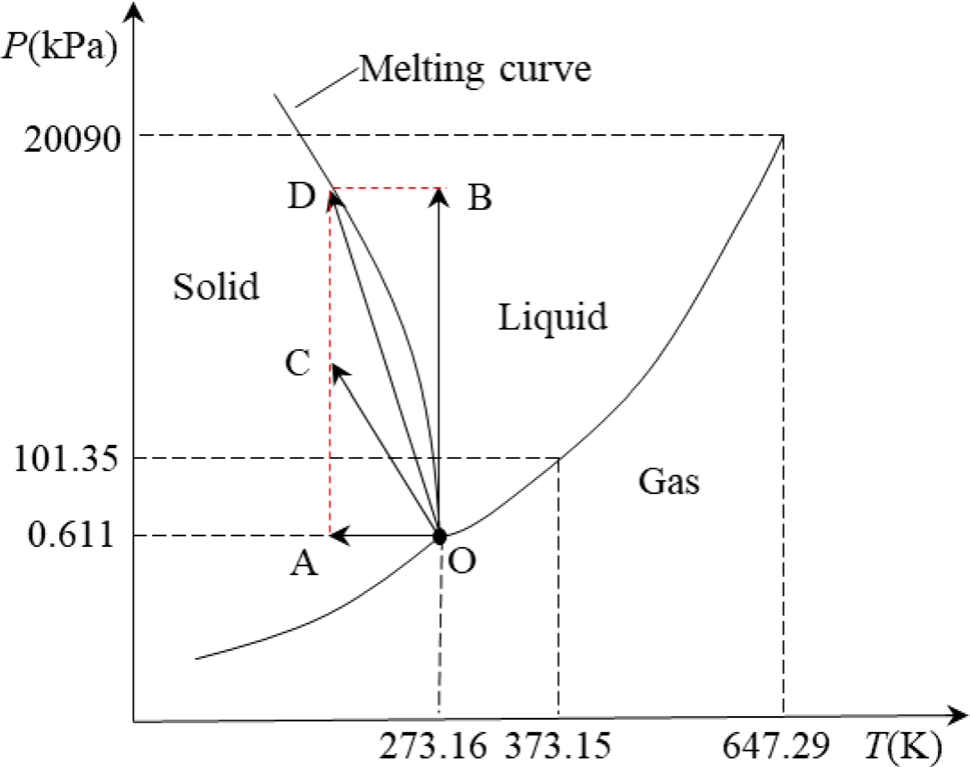
Three-phase graph of water.

Equation (4) shows that when the temperature changes, the pressure will change accordingly to establish a new equilibrium, so that the ice and water phase change is in a state of dynamic equilibrium. The ice and water phase transition evolution processes are shown in Fig. 4. The curves in the figure are the phase boundary, and point O is the three-phase turning point. At O point, the three states of water can exist simultaneously. The solid–liquid phase change point of water at different pressures can be calculated from the transformation of Eq. (4), as shown in Eq. (5) ^42^, and the results are shown in Table 2.

5 $$ \int_{{T_{0} }}^{T} {\frac{{\Delta H_{fus} }}{{T^{2} }}} dT = \left( {V_{i} - V_{w} } \right)\left( {P - P_{0} } \right), $$

where $\Delta H_{fus}$ is the enthalpy change of melt at temperature *T*; $V_{i} - V_{w}$ is the molar volume change during icing, m^3^/mol, which is the product of the molar mass and the density derivative; $P_{0}$ is the standard atmospheric pressure ($P_{0}$ = 0.1 MPa); and $T_{0}$ is the freezing point of free water ($T_{0}$ = 273.15 K).

**Table 2:** Solid–liquid phase transition point of water.

*P *(kPa)	0.611	59.8 × 10^3^	110.4 × 10^3^	156.0 × 10^3^	193.5 × 10^3^
*T *(℃)	0.01	− 5.0	− 10.0	− 15.0	− 20.0

Figure 4 and Table 2 show that the freezing process has the following stages: isobaric cooling stage (OA) → boosting pressure cooling stage (OC) → boosting pressure cooling stage (OD). The melting process has the following stages: pressure reduction and warming phase (CO) → isobaric warming stage (AO). As the freezing temperature decreases, the pore pressure gradually increases, and the freezing point of the water decreases. Two-phase equilibrium is reached when the speeds of water freezing in the system and ice melting into water are the same. That is, the freezing point of the water in the system is decreasing and the unfrozen water content will not change.

### Characteristics of unfrozen water content changes during freeze–thaw

**Figure 5:**
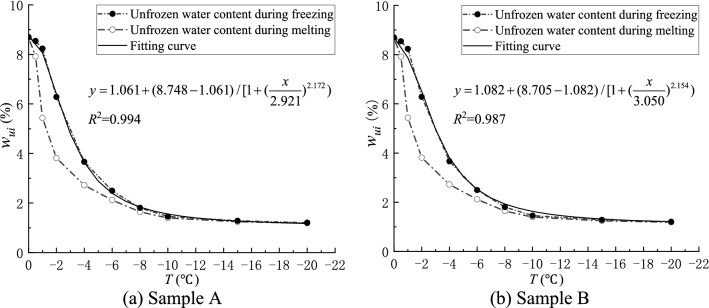
Relationship between unfrozen water content and temperature during coal sample freeze–thaw.

The relationship between the unfrozen water content and temperature in the freezing and melting process is shown in Fig. 5. The unfrozen water content in the process of melting at the same temperature is lower than that in the process of freezing. Especially in the range of 0 °C to − 1 °C, the unfrozen water content in the process of freezing and thawing varies greatly, which is called the hysteresis phenomenon.

Figure 5 shows that in the freezing process, the unfrozen water content is not obvious in the range of 0 °C to − 1 °C, and the pore water did not undergo the ice-water phase change, which is called the super-cooling phenomenon. In the range of − 1 °C to − 6 °C, the unfrozen water content dropped sharply. Below − 6 ℃, the unfrozen water content gradually tended to be stable with the reduction of freezing temperature. At this time, the unfrozen water was mainly the film water on the pore surface and the pore water in the micro-pore; the thickness of the film water gradually decreased with the reduction of the freezing temperature, becoming increasingly difficult to freeze, until the thickness of the film water stabilized^43^. The pore water with large pores also froze gradually with the dropping freezing temperature, but a part of the pore water always remained unfrozen^44^, so the unfrozen water content gradually stabilized with the reduction of temperature. The change trend of unfrozen water content in the melting process is different from that in the freezing process. As the temperature is close to 0 ℃, the pore water content during the melting process increased with the temperature until the ice was completely melted, and no super-cooling phenomenon occurred in the melting process.

The unfrozen water content of the coal shows a hysteresis phenomenon during the melting process. One of the main reasons is that the freezing point of water in the freezing process and the melting point of ice in the melting process are inconsistent at the same temperature, and the freezing point of water varied with the pressure. Given that coal is a porous medium, water is mainly distributed in the pores of the coal. At the early stage of freezing, the ice-water phase change in the pore space caused the pressure in the pore space to increase sharply, causing the pore water freezing point to decrease sharply. However, the pore pressure gradually decreased during the melting process until the pore pressure returned to normal pressure and the melting point of the ice rose to 0 °C. That is, at the same temperature, the pore pressure in the freezing process is higher than the pore pressure in the melting process, and the freezing point of water in the freezing process is lower than the melting point of ice in the melting process.

**Figure 6:**
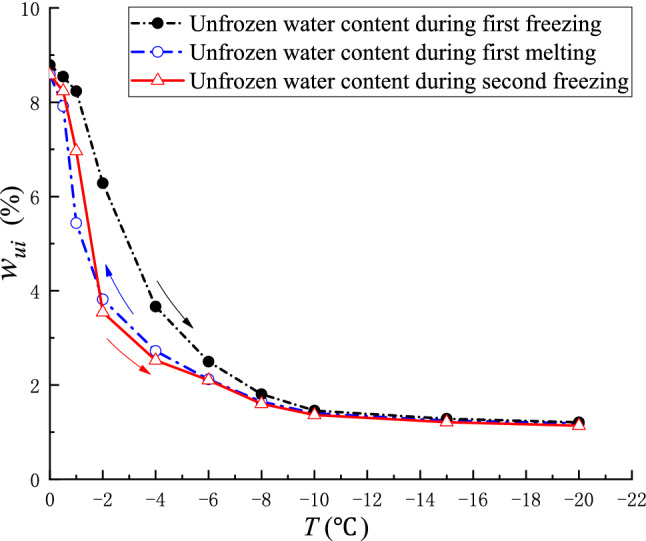
Relationship between unfrozen water content and temperature of coal sample C during freeze–thaw.

Another important cause of the inconsistency between the freezing point of water during freezing and the melting point of ice during melting at the same temperature was the change in the pore radius of the coal mass due to the phase change of ice and water during the freeze–thaw process. In order to verify the influence of pore changes on the freezing point and melting point, the second freezing test was carried out on sample C. Using the unfrozen water content calculation formula, the unfrozen water content curve of coal sample C in the negative temperature-freezing region is obtained (Fig. 6). As shown in Fig. 6, the unfrozen water content in the second freezing process is closer to the first melting process. The volume expansion caused by the water phase change during the freezing process led to an increase in the pore radius of the coal mass, which in turn increased the total volume of the pore. The pore volume increased in the freezing process and basically remained constant in the absence of external pressure. Thus, the overall pore volume was larger than that before freezing, while the overall content of ice and water in the freeze–thaw process was the same. Therefore, at the same temperature, the proportion of ice and water volume in the melting process was smaller than in the freezing process, and the pore pressure in the melting process was smaller than that in the freezing process. According to the Gibbs–Thomson equation, as the pores increase, the melting point of pore ice will increase, so the freezing–thawing characteristic curve of coal will show obvious hysteresis.

## Pore structure and pore water freezing: criticality analysis

### Pore structure analysis of coal mass structure

The NMR $T_{2}$ and pore distributions have a certain conversion relationship. Therefore, the characterization of the pore volume of the coal mass can be achieved by the NMR method. The surface relaxation time of the coal mass is determined by the relaxation effect of the coal mass pore space and water. In the state of water saturation, the larger the specific surface area of the coal mass pore is, the larger the surface relaxation time is. The relationship can be expressed by Eq. (6)^45^:

6 $$ \frac{1}{{T_{2} }} \approx \rho_{2} \frac{S}{V} = \rho_{2} \frac{F}{r}, $$

where $\rho_{2}$ is the relaxation strength of the transverse surface, m/ms; $F$ is the geometry factor (3 for spherical pore space and 2 for columnar pore space); and $r$ is the pore radius, m.

From Eq. (6), the pore radius can be expressed as

7 $$ r = T_{2} \rho_{2} F. $$

**Figure 7:**
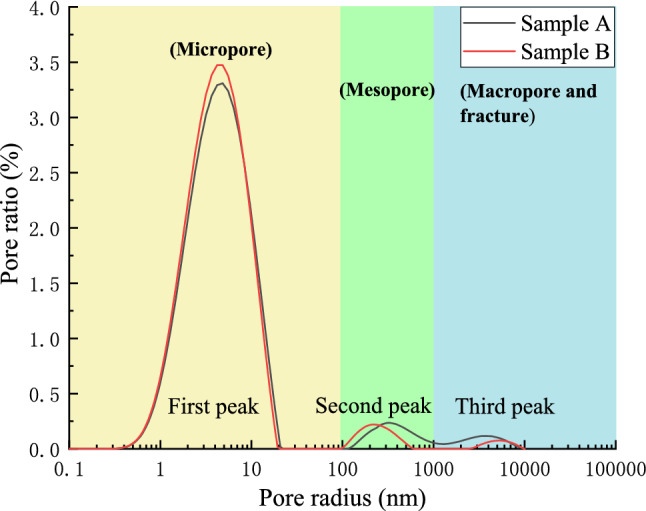
Pore radius distribution of coal sample.

The surface relaxation coefficient of substance in Eq. (7) changes with the coal rock properties, and the experimental coal sample is anthracite. According to Xie et al.^46^, the relaxation strength of the transverse surface $\rho_{2}$ of a high rank coal can be taken as 0.54 × 10^–8^ m/ms. The pores in the coal rocks are mostly cylindrical, so geometry factor *F* takes the value of 2. The pore radius distribution of the experimental coal samples is calculated using Eq. (7), as shown in Fig. 7.

According to the sizes of the coal pores classified by Yao et al.^47^ and Li et al.^48^, the first, second, and third peaks in Fig. 7 respectively correspond to the micropores, mesopores, and macropores and fractures in the coal. Figure 7 indicates that the peak fluctuation of the two coal samples is similar. Most of the pore diameters ranged from 0.2 to 20 nm, excluding the large fractures. The specific data of the proportion of each peak value of pore diameter distribution are shown in Table 3.

**Table 3:** Percentage ratio of each peak in pore size distribution.

Coal sample	Peak number	Peak starting radius	Peak end radius	Peak ratio
A	1	0.217	20.734	93.318
	2	117.639	1336.003	4.67
	3	1336.003	10,723.363	2.012
B	1	0.217	19.867	96.259
	2	109.742	1012.056	3.093
	3	2795.548	10,956.154	0.649

Figure 7 and Table 3 show that the first peak area of the pore diameter distribution of the two coal samples is relatively large, as the pore volume of the two experimental coal samples with a pore diameter below 20 nm accounted for 93.318% and 96.259% of the total pore and fracture volumes, respectively. The second and third peaks represent the proportions of the pore and fissure volumes with a pore diameter above 10^2^ nm, accounting for 6.682% and 3.742%, respectively. The small pores in the Jiulishan Coalmine samples are well-developed, while the large fractures and pores are poorly developed.

### Law of pore water distribution during freeze–thaw

**Figure 8:**
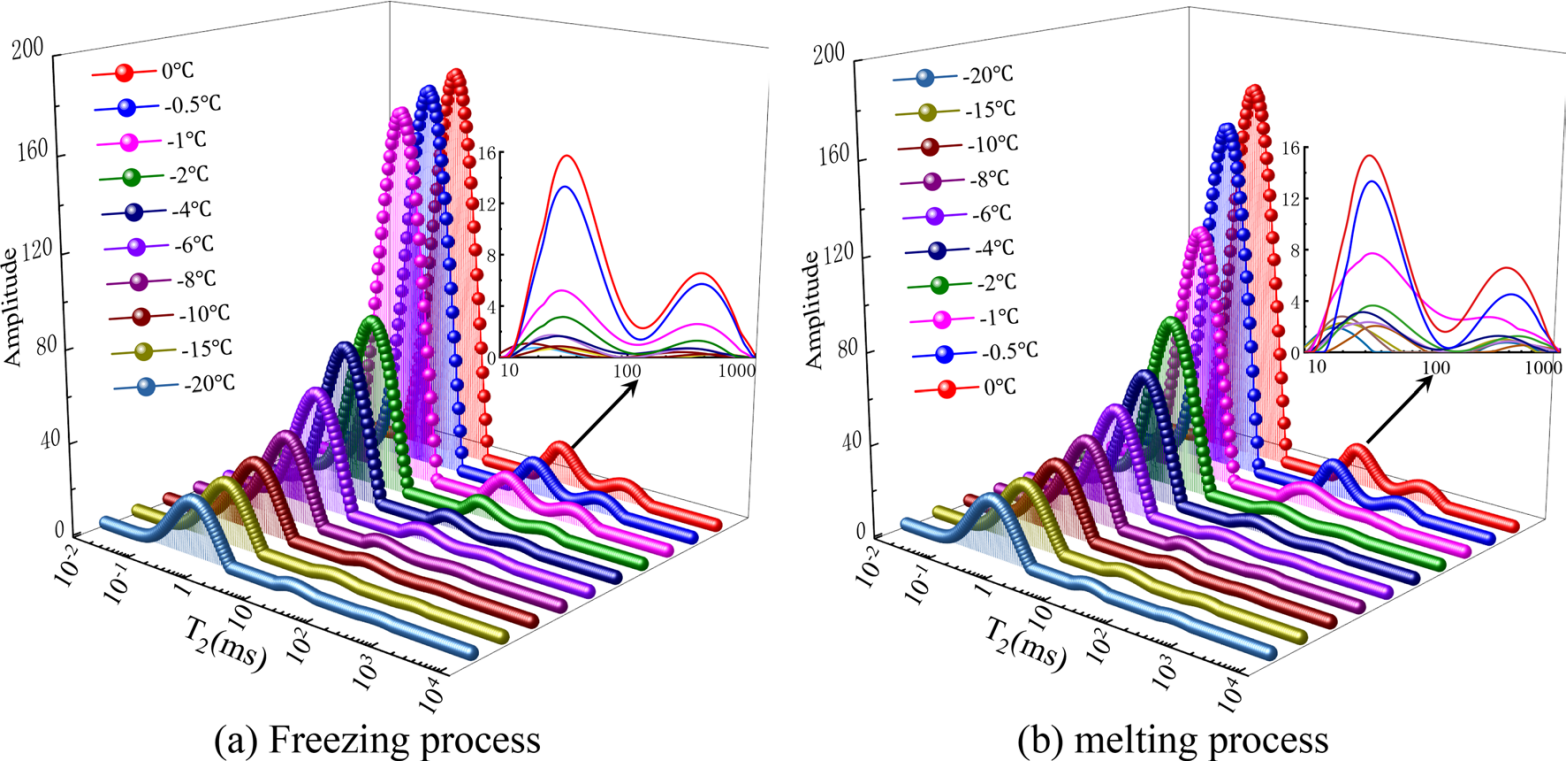
*T*_2_ distribution curve of coal sample A during freeze–thaw.

Equation (7) indicates that $T_{2}$ is proportional to the pore radius. That is, the larger the $T_{2}$ value is, the larger the radius of the pore is and where the pore water is located, and vice versa. Therefore, the peak area enclosed by the $T_{2}$ distribution curve and the abscissa axis can characterize the amount of pore water in the sample^49^. Given the similarity of the $T_{2}$ distribution law of the experimental coal samples and the space limitation of this paper, coal sample A is taken as an example to analyze the pore water distribution of the coal samples in the freeze–thaw process. The $T_{2}$ distribution curve of coal sample A during freeze–thaw at different temperatures is shown in Fig. 8.

Figure 8a shows the $T_{2}$ distribution curve of the temperature of coal sample A during the freezing process. When the freezing temperature decreased from − 0.5 to − 1 ℃, the area of the second and third peaks decreased abruptly, while the area of the first peak decreased, but the decrease was not large, indicating that the water in the large and medium pores of the coal sample was frozen, and the water in the micro pores was not significantly frozen. When the freezing temperature decreased from − 1 to − 2 ℃, the area of the first peak began to decrease significantly, and the area of the second and third peaks also decreased with the temperature, indicating that the water in the large and small pore spaces of the coal sample in this section of the interval were frozen. When the freezing temperature decreased from − 2 to − 10 °C, the area of the first peak continued to decrease, and the area of the second and third peaks changed less significantly. In this interval, the water in the large pore space is frozen. When the freezing temperature decreased from − 10 to − 20 ℃, the area of the three peaks decreased with the temperature but was nearly stable.

Figure 8b shows the $T_{2}$ distribution curve of the temperature of coal sample A during the melting process. When the temperature rose from − 20 to − 2 ℃, the area of the first peak increased continuously, while the area of the second and third peaks changed less obviously, indicating that the main reason in this section is the continuous melting of ice in the micro and small pores in the coal sample. When the temperature rose from − 2 to − 0.5 °C, the area of the second and third peaks became significantly larger, indicating that the ice in the large and medium pores in this section began to melt continuously.

In summary, in the freezing process of the coal sample, the pore water in the large pore begins to freeze first. In the melting process, the ice in the small pore begins to melt first, and the temperature increases to a certain degree before the ice in the large pore begins to melt. Figures 7 and 8 also shows that the unfrozen water in the pore space was mainly distributed in the pore space with a radius of 10 nm or less.

### Relationship between pore water freezing point and pore radius in coal

The freezing point of pore water in coal is mainly influenced by the pore size. The smaller the pore is, the lower the freezing point of the water is. Based on thermodynamic theory, the relationship between the freezing point of pore water and the pore radius can be expressed by Eq. (8)^50^:

8 $$ \ln \left( {\frac{{T_{r} }}{{T_{0} }}} \right) = - \frac{{F\sigma_{i - w} M_{r} }}{{\rho_{w} r_{m} Q}}, $$

where $T_{r}$ is the freezing temperature of water in pores with diameter of $r$, K; $T_{0}$ is the freezing temperature of water at atmospheric pressure, K; $\sigma_{i - w}$ is the ice–water interface tension, dyne/cm; $M_{r}$ is the molecular weight of water, g/mol; $\rho_{w}$ is the density of water, g/cm^3^; $r_{m}$ is the pore radius, cm; and $Q$ is the dissolved heat of water, kJ/mol K.

Surface tension is measured by using drop volume method, which is expressed as:

9 $$ \sigma = \frac{v\rho g}{{2\pi r}}, $$

where $v$ is the droplet volume, which is a constant (0.07 cm^3^); $\rho$ is the liquid density, g/cm^3^; $g$ is gravitational acceleration, 980 cm/s^2^; and $r$ is the dropping tip radius, cm.

From Eq. (9), the interface tension is calculated as

10 $$ \sigma_{i - w} = \frac{{v\left( {\rho_{w} - \rho_{i} } \right)g}}{2\pi r}. $$

By substituting Eq. (10) to Eq. (9), the pore water freezing point is calculated as follows:

11 $$ T_{r} = T_{0} \cdot \exp \left[ { - \frac{{FM_{r} v\left( {\rho_{w} - \rho_{i} } \right)g}}{{2\pi r\rho_{w} r_{m} Q}}} \right]. $$

**Figure 9:**
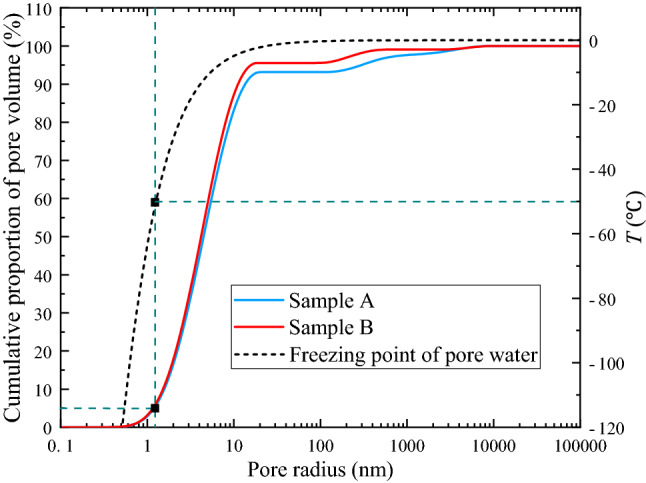
Relationship between pore radius and pore water freezing point.

In Fig. 9, the black dashed line shows the relationship between the freezing point and the pore radius, and the blue and red lines are the cumulative proportions of the pore volumes of the two coal samples below a given pore diameter. The freezing point of pore water gradually decreased as the pore radius decreasing, and the freezing point decreases rapidly when the pore radius reached 10 nm. The pore radius ranged from 0.1 to 10 nm, and the freezing point of pore water sharply dropped with the decrease of the pore radius. As shown by the blue dashed line in the Fig. 9, for pores with a pore radius below 1.54 nm, the cumulative volume accounted for 5%. At this time, the freezing point of the pore water was − 50 ℃. Thereafter, the freezing point of water decreased with the pore radius at a rate of up to 600 °C/nm.

## Conclusion

In this study, it was shown that the liquid water content inside the coal can be measured by the NMR technique, and the pore structure of coal can also be obtained from the $T_{2}$ spectra. The fugacity of unfrozen water in coal and the change in the pore water freezing point are obtained by NMR experiments on saturated water coal during the freeze–thaw process. This study draws the following conclusions.

1. In the freezing process, the water in the pores and fractures of the coal mass undergoes three stages: isobaric cooling, boosting pressure cooling, and boosting pressure cooling phase equilibrium. After reaching the phase equilibrium, the freezing point of the water in the system continues to decrease, and the unfrozen water content remains constant. In the melting process, the temperature change prevents phase equilibrium, going through the phases of pressure reduction warming and isobaric warming. Hysteresis occurs in the unfrozen water content during the melting process, and the unfrozen water content is higher during the freezing process than during the melting process at the same temperature.
2. According to theoretical calculations and analysis, the pore size distribution of the coal mass shows that the small pore volume of the coal samples from Jiulishan Coalmine is well-developed, while the large pores and fractures are poorly developed. The pores with a pore radius range of 0.1–10 nm account for more than 90%, while the pores and fractures with a pore radius above 10^2^ nm account for a relatively small proportion.
3. The unfrozen water is mainly the film water on the pore surface and the pore water with a pore radius below 10 nm when the coal mass freezes to equilibrium. When the coal sample freezes, the pore water in the large pore begins to freeze first. When it melts, the ice in the small pore begins to melt first, and the temperature increases to a certain degree before the ice in the large pore begins to melt.
4. The freezing point of pore water decreases gradually with the decrease of the pore diameter. When the pore radius decreases to 10 nm, the freezing point of pore water starts to decrease sharply with the decrease of the pore diameter. When the pore radius is 1.54 nm, the freezing point of pore water changes at a speed of 600 °C/nm.
